# Recovery of coral assemblages despite acute and recurrent disturbances on a South Central Pacific reef

**DOI:** 10.1038/s41598-018-27891-3

**Published:** 2018-06-26

**Authors:** Mehdi Adjeroud, Mohsen Kayal, Claudie Iborra-Cantonnet, Julie Vercelloni, Pauline Bosserelle, Vetea Liao, Yannick Chancerelle, Joachim Claudet, Lucie Penin

**Affiliations:** 1 Institut de Recherche pour le Développement (IRD), UMR 9220 ENTROPIE, UPVD 52 avenue Paul Alduy, 66860 Perpignan, France; 2 Laboratoire d’Excellence CORAIL, Perpignan, France; 3 USR 3278 CNRS-EPHE-UPVD, Centre de Recherches Insulaires et Observatoire de l’Environnement, UPVD 52 avenue Paul Alduy, 66860 Perpignan, France; 4 Global Change Institute, The University of Queensland, Brisbane, QLD 4072 Queensland Australia; 5 ARC Centre of Excellence for Coral Reef Studies, The University of Queensland, Brisbane, QLD 4072 Queensland Australia; 6 Pacific Community (SPC), Fisheries, Aquaculture and Marine Ecosystem division, BP D5, 98848 Noumea, New Caledonia; 7 National Center for Scientific Research, PSL Université Paris, CRIOBE, USR 3278 CNRS-EPHE-UPVD, Maison des Océans, 195 rue Saint-Jacques, 75005 Paris, France; 8 Université de la Réunion, UMR 9220 ENTROPIE, 15 avenue René Cassin CS 92003, 97744 Saint Denis, Cédex 9, La Réunion France

## Abstract

Coral reefs are increasingly threatened by various types of disturbances, and their recovery is challenged by accelerating, human-induced environmental changes. Recurrent disturbances reduce the pool of mature adult colonies of reef-building corals and undermine post-disturbance recovery from newly settled recruits. Using a long-term interannual data set, we show that coral assemblages on the reef slope of Moorea, French Polynesia, have maintained a high capacity to recover despite a unique frequency of large-scale disturbances which, since the 1990s, have caused catastrophic declines in coral cover and abundance. In 2014, only four years after one of the most extreme cases of coral decline documented, abundance of juvenile and adult colonies had regained or exceeded pre-disturbance levels, and no phase-shift to macroalgal dominance was recorded. This rapid recovery has been achieved despite constantly low coral recruitment rates, suggesting a high post-disturbance survivorship of recruits. However, taxonomic differences in coral susceptibility to disturbances and contrasting recovery trajectories have resulted in changes in the relative composition of species. In the present context of global coral reef decline, our study establishes a new benchmark for the capacity of certain benthic reef communities to sustain and recover their coral cover from repeated, intense disturbances.

## Introduction

Coral reefs are threatened by various types of local and large-scale anthropogenic and natural disturbances that cause widespread mortalities of scleractinian corals, the primary framework builders and key components of reef health and biodiversity^1–3^. Many reefs have undergone striking phase shifts in their benthic communities, which classically involves the replacement of corals by fleshy macroalgae or other non reef-building organisms, an undesirable state which provides less ecosystem goods and services^4^. Given the likely continuation of such disturbances in the next decades, coral reefs are expected to be highly vulnerable to future environmental changes^5–8^. In this context, understanding the determinants of coral recovery and identifying disturbance regimes that cause shifts in species composition is crucial for the preservation of reef ecosystems.

While coral reefs from various regions have undergone severe and likely irreversible degradation^9,10^, some reefs have shown the capacity to recover from severe disturbances^11–13^. Recovery of coral populations following disturbances relies on the arrival of newly settling larval recruits, as well as on the growth and propagation of surviving coral colonies^13,14^. Given the slow growth and long life span of most coral species, understanding mechanisms of coral recovery requires long-term surveys of population dynamics and demography^12,15,16^. However, due to the difficulty in collecting such data over a time-period that allows for coral decline-recovery cycles to occur, few studies have examined the interplay between early recruitment, dynamics of juvenile and adult populations, and disturbances at a relevant time-scale^15^. Consequently, there is still a critical knowledge gap in the ecological drivers of coral community trajectory, particularly during recovery processes that follow disturbances^16,17^.

**Figure 1:**
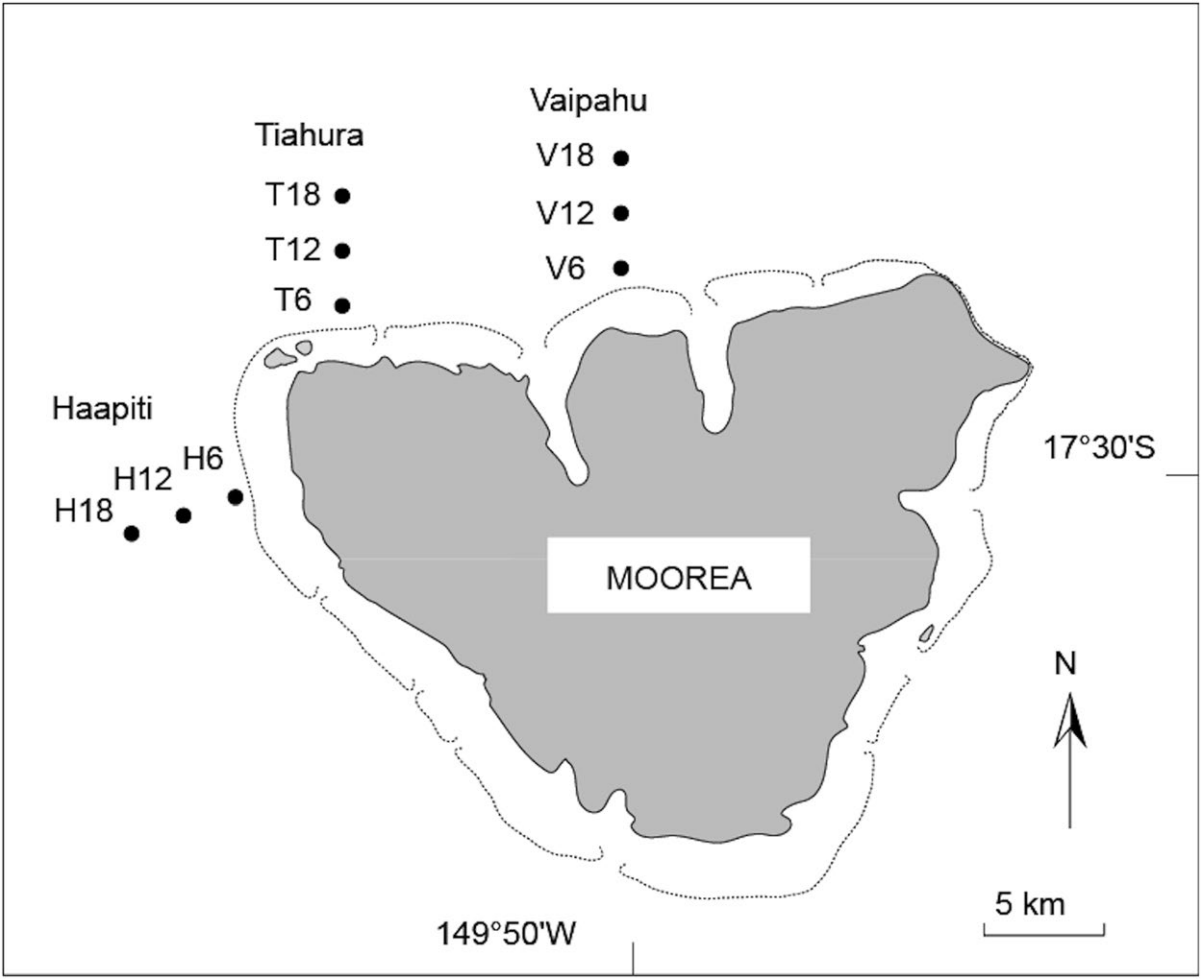
Location of the nine stations sampled on the outer reef slope around Moorea, representing three locations (Vaipahu, Tiahura, Haapiti), and three depths (6, 12, and 18 m). Stations codes are abbreviated as follows: the first letter indicates the location (V: Vaipahu; T: Tiahura; H: Haapiti), and the associated number represents the depth (6, 12, and 18 m). Dashed lines represent the approximate extent of the reef front surrounding Moorea. Note that the position of the stations is provided schematically and does not follow the spatial scale of the map. This map was created using Adobe Illustrator CS5 (http://www.adobe.com/fr/products/illustrator.html).

Here, we analyzed a unique long-term dataset to examine the interannual variability in cover (1991–2014; at a reference site) and abundance (2003–2014; across different locations and depths) of the three benthic stages of corals (recruits, juveniles, and adults)^18^ on the outer reef slope of Moorea Island (17°30′S, 149°50′W) in French Polynesia (Fig. 1). Since the 1980s, this reef has been affected by a remarkably high frequency and intensity of the three most destructive large-scale disturbances that affect Indo-Pacific reefs^10,19^: coral bleaching events, cyclones, and outbreaks of the coral-killing crown-of-thorns seastar *Acanthaster* spp. (COTS). Moorea is thus a unique study site to examine the temporal dynamics and recovery trajectories of coral populations affected by acute and recurrent disturbances, and the data set analyzed here is one of the most comprehensive in the Indo-Pacific.

## Results and Discussion

**Figure 2:**
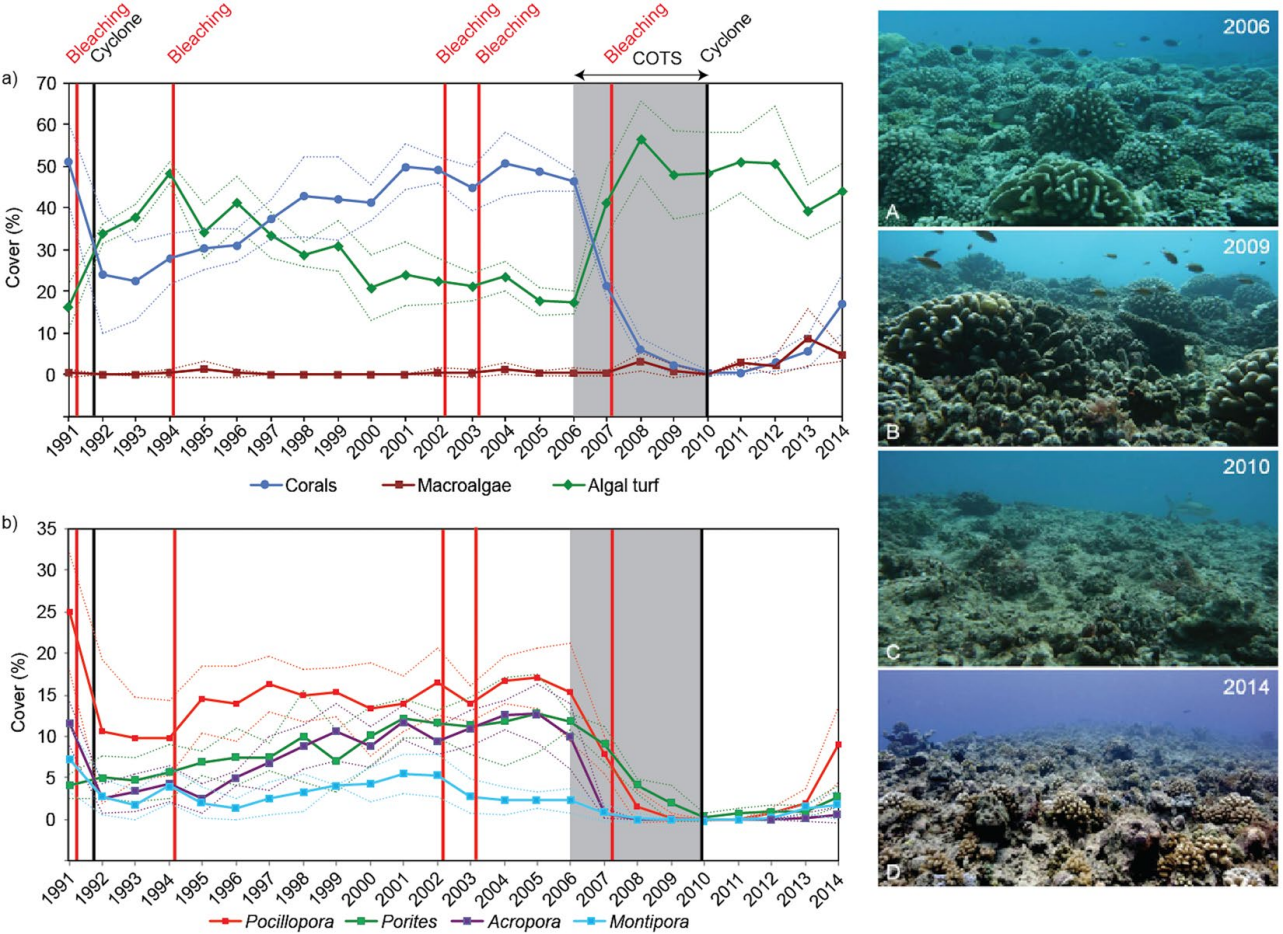
Impact of eight large-scale disturbances between 1991 and 2014 on (**a**) cover of corals (all 18 genera), macroalgae and turf algae, and (**b**) cover of the four dominant coral genera at 12 m depth at Tiahura reef, Moorea island. Dotted lines represent standard deviation. A portion of the reef is shown through time (photos A–D). (A) Coral dominate the healthy reef (coral cover >45%). (B) Turf algae have colonized dead coral colonies following outbreaks of the coral-killing crown-of-thorns seastar *Acanthaster* spp. (COTS) (<5% coral cover). (**C**) Mostly dead and weakened coral colonies were swept away by the cyclone Oli in February 2010 (<1% coral cover). (D) Juvenile colonies of Pocillopora spp. have recolonized the substrate (∼17% coral cover). © Photos Mohsen Kayal (A–C), Yannick Chancerelle (D).

Prior to the establishment of our survey in 1991, Moorea’s outer reefs had already recovered from two mass bleaching events (1984 and 1987), and a COTS outbreak (1980–1982) that reduced coral cover from ∼45% in 1979 to ∼12% in 1982^20^. Between 1991 and 2014, the reefs were impacted by two cyclones, five bleaching episodes (associated with high sea-surface temperatures), and a catastrophic COTS outbreak that caused two additional episodes of major coral decline (Fig. 2a). The first major decline resulted from the combined impacts of a cyclone and a bleaching event that reduced live coral cover from ∼51% in 1991 to ∼22% in 1993. Coral cover increased rapidly after 1993 and recovered pre-disturbance values of 45–50% that persisted from 2001 to 2006, despite subsequent bleachings in 1994, 2002, and 2003 resulting from thermal anomalies that were similar in extent and prevalence to the 1991 event, but did not cause mass mortality^21^. The second major episode of coral decline resulted from the successive occurrence of a COTS outbreak in 2006–2010 followed by a cyclone in February 2010. This constituted one of the most extreme cases of coral reef decline documented, with live coral cover decreasing from ∼49% in 2005 to <1% in 2010. Coral cover increased gradually thereafter, reaching ∼17% in 2014 (Fig. 2a; Supplementary Table S1), and has since recovered to pre-disturbance levels (∼50%) at several reef slope locations in 2016^22^.

Both major coral decline events were accompanied by a rapid increase in turf algae (from ∼16% to ∼50% cover; Fig. 2a; Supplementary Table S2), which is generally among the first to colonize vacant reef space^4^. However, in contrast with the ‘typical’ phase shifts that are increasingly reported on coral reef ecosystems^4^, a consecutive proliferation of fleshy macroalgae was not observed at Moorea (Fig. 2a; Supplementary Table S3). Resistance of Moorea’s reefs to a transition to macroalgal dominance probably results from the high grazing pressure by herbivorous fish, most notably the parrotfishes (Scarinae), whose densities remain stable, or even increase, following disturbances at Moorea, and which are considered well above those needed to prevent proliferation of macroalgae^23,24^.

The response of coral populations to disturbances and their recovery trajectories differed among the four dominant genera (Fig. 2b), which represent >80% of the overall coral cover. *Acropora*, *Montipora* and *Pocillopora* cover dropped following the disturbances of 1991 (∼ two-fold decrease), but between 1992 and 2005, *Acropora* showed a relatively continuous recovery trend, *Montipora* declined further as a result of the bleaching events of 1994 and 2002, and *Pocillopora* recovered partially within four years and remained relatively stable (13–17%) thereafter. In contrast, the cover of *Porites* was unaffected by the first five disturbances, and its cover increased gradually from ∼4% to ∼12% between 1991 and 2005 to become the second most abundant taxa. Since the second major coral decline (2006–2010), which affected all of the four genera, recovery has been mostly driven by *Pocillopora*, whose percent cover had increased rapidly from 2010 to 2014 (from ∼0% to ∼9%).

**Figure 3:**
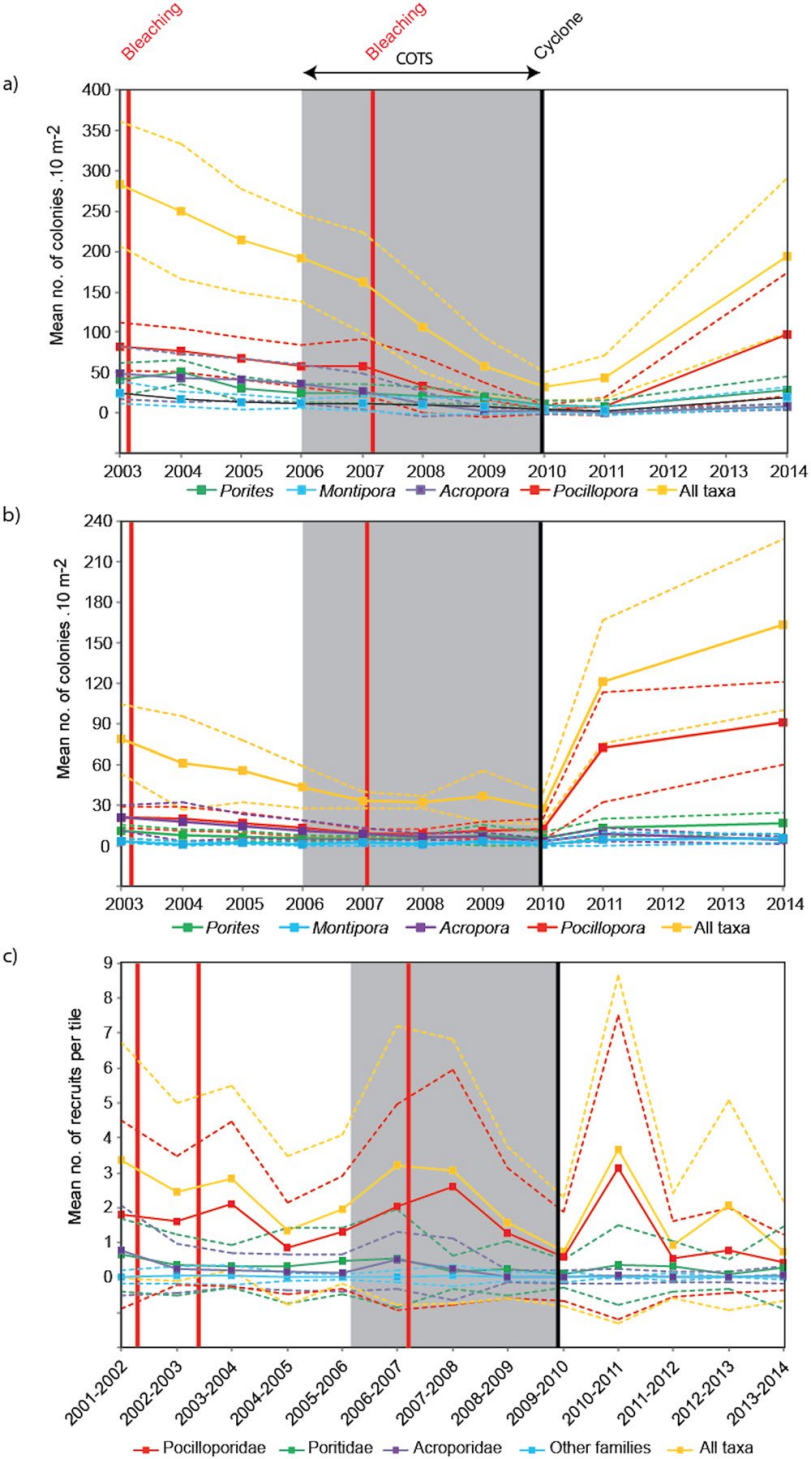
Temporal dynamics in (**a**) adult, (**b**) juvenile, and (**c**) recruit coral abundance impacted by recent disturbances. All three locations and three depths sampled around Moorea were pooled. Dotted lines represent standard deviation.

Similar to changes in percent live coral cover, a general decreasing trend in the abundance of juvenile and adult coral colonies was observed between 2003 and 2010 (∼ ten-fold decrease for adults), quickly followed by a six-fold increase between 2010 and 2014 (Fig. 3a,b). This pattern was recorded at all locations and depths, even if the trajectories varied among coral taxa and life stages (see generalized linear mixed-effect model; Supplementary Tables S4 to S6). Adult coral trajectories varied with depth for all genera except *Acropora*, which differed among locations (see Supplementary Figs S1 to S5). Juvenile coral trajectories were consistent across depths and locations for *Pocillopora* and *Montipora* but varied with depths for *Porites* and with locations for *Acropora* (see Supplementary Figs S6 to S10). The post-disturbance increase in the overall juvenile and adult abundance was mainly driven by *Pocillopora* and, to a lesser extent, *Porites*. In contrast, no signs of recovery were found for *Acropora* juveniles and adults at any locations or depths. The temporal dynamics of coral recruits were different to those described for adults and juveniles (Fig. 3c). For recruits, no obvious trend was recorded during the monitoring period (2001–2014), except an overall decreasing trajectory of Acroporidae, and a slight increase of Pocilloporidae at 6 m depth at Vaipahu after the occurrence of cyclone Oli in 2010 (see Supplementary Figs S11 to S15). In fact, recruitment rates at Moorea were always low (<140 recruits.m^−2^) compared to other Pacific reefs characterized by a dominance of Pocilloporidae and a low contribution of Acroporidae recruits, similar to those of high-latitude or sub-tropical Indo-Pacific reefs^17,25^.

Moorea is among the few Indo-Pacific reefs that have shown the capacity to recover from severe and recurrent large-scale disturbances^12,26^. However, the post-disturbance dynamics of coral assemblages reported here are marked by a number of striking differences compared to those dominating contemporary literature. Notably, despite the frequency and severity of the disturbances that have affected Moorea in the last three decades, and the two major declines in coral cover and abundance that rival some of the biggest losses reported elsewhere^9^, this reef has shown, on two occasions, a remarkable capacity to quickly recover. Furthermore, we found successful recovery at all habitats surveyed, across various depths and locations. Successful recovery is generally associated with significant inputs of coral recruits^27,28^, but at Moorea, recovery was achieved despite constantly low recruitment rates. Larval input from other less impacted islands may explain this successful recovery, as suggested by the strong genetic connectivity among populations of *Pocillopora meandrina*, the dominant species on the outer reef slope within the Society Archipelago^29^. Alternatively, coral populations in habitats less impacted by disturbances, such as deeper reef slopes (>30 m depth) and lagoons, may produce enough larvae to support recolonization through self-seeding^30,31^. Moreover, the recruits that colonized the reefs after the 2006–2010 decline have probably benefited from a higher availability of suitable substrate, with a low level of macroalgal cover due to the high grazing pressure by herbivorous fish, and a lower post-settlement mortality due to reduced predation and competition with other benthic organisms^18,19,32^.

**Figure 4:**
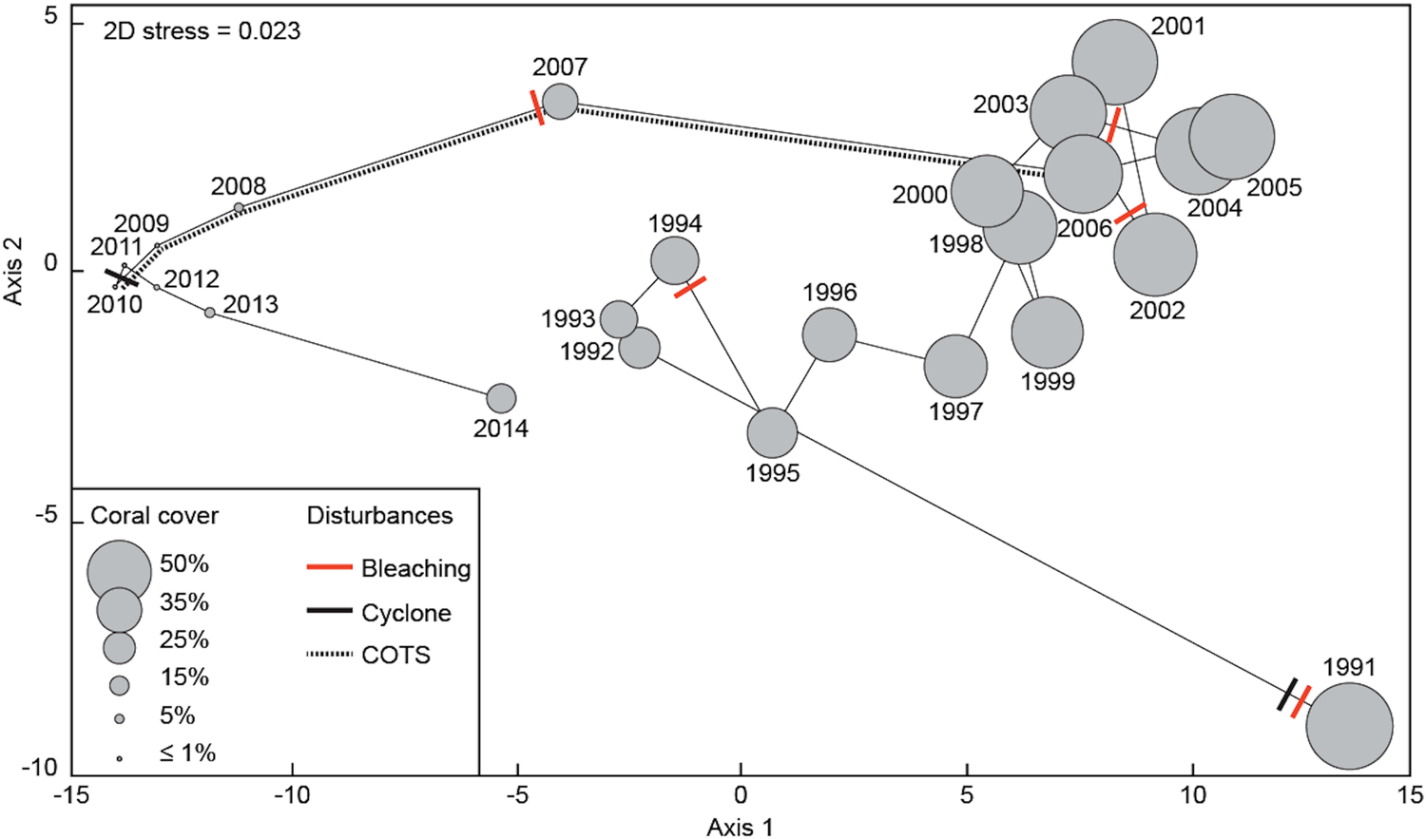
Changes in the structure of the coral assemblage from 1991 to 2014 at 12 m depth at Tiahura reef. Non-metric multidimensional scaling (nMDS) using the Bray-Curtis dissimilarity index of the cover of coral genera recorded annually. The scale of the circle for each year is proportional to the overall coral cover. Vectors link points by chronology.

Our results highlight the contrasted susceptibility among coral taxa to disturbances and recovery trajectories that led to significant changes in the structure of the assemblages between 1991 and 2014. This time-interval was characterized by short periods of intense reorganization in response to the disturbances of 1991 and 2006–2007, followed by longer periods of slower changes (Fig. 4). This outcome is in accordance with the process of ‘recovery without resilience’^20^, highlighting that successful recovery in terms of overall coral cover and abundance is not necessarily accompanied by a reassembly of the relative abundances of component taxa, thus preventing a ‘true’ and complete recovery. These discrepancies in the capacity of coral taxa for population maintenance and recovery are most likely linked to differences in life history traits such as reproduction strategies, stress tolerance, growth capacities, and competitive abilities^33,34^. Indeed, *Pocillopora* clearly exhibits an opportunistic life strategy in French Polynesia, with a high population turnover resulting from comparatively higher recruitment rates, a high potential for larval dispersal and colonization, fast growth, and low resistance to perturbations^18,34^. On the other hand, massive *Porites* shows a resistant life strategy, with slow turnover among individuals resulting from the production of fewer off-spring endowed with a high capacity for survival, slow growth to large colony sizes, as well as lower rates of decline in presence of harsh conditions and catastrophic events^18,34^. *Acropora* and *Montipora* are competitive species characterized by high aptitudes to preempt space and resources in optimal environments through faster individual growth and by a high susceptibility to disturbances^19,34^. A high potential for recovery following physical disturbance, such as wave damage during cyclones, has also been observed for *Acropora*^11^, which has been related to the capacity of this taxon for asexual propagation through high survival and reattachment of colony fragments^34^. However, recurrent disturbances have probably overwhelmed the capacity of competitive coral taxa for recovery, and have progressively pushed communities towards the predominance of opportunistic and stress-tolerant species. At Moorea, *Pocillopora* and *Porites* are thus clearly the contemporary and, most probably, future ecological ‘winners’, whereas *Acropora* and *Montipora* appear to be the ‘losers’^32,34^.

The outcomes of this study support the hypothesis that, regardless of their ultimate fate, some reefs will undergo gradual changes in the structure of their coral communities in response to major stress, rather than collapse abruptly and irreversibly (see the concept of ‘novel coral reef ecosystems’^35^). However, successful recovery is conditioned by a low frequency of large-scale disturbances that should allow juvenile corals to reach maturity and contribute to the larval pool, but this scenario is challenged by some recent projections^6,8^. Another critical point now is to determine if changing coral assemblages will maintain the ecological and socio-economic goods and services that they have provided until recently^5^.

## Methods

### Study site

This study was conducted at Moorea (17°30′S, 149°50′W), located 17 km to the northwest of Tahiti in the Society Archipelago, French Polynesia. Approximately 17000 people live on Moorea. It comprises 134 km^2^ of land, 49 km^2^ of reefs and lagoon, and has a circumference of 61 km (Fig. 1). The island is surrounded by a narrow coral reef of 2 km maximum width, which can be divided into three major reef habitats: fringing reef, barrier reef flat (separated from the fringing reef by a narrow sandy channel), and outer reef slope (separated from the barrier reef flat by the reef front). The reef slope is largely free of direct anthropogenic disturbances and nutrient loads are low^36,37^. The tides are semi-diurnal with an amplitude rarely exceeding 40 cm. Surveys of coral abundance were undertaken on the outer reef slope in three water depths (6, 12, and 18 m) at each of three locations (Vaipahu, Tiahura, Haapiti), giving a total of nine stations (Fig. 1). Vaipahu and Tiahura are situated on the northern shore subjected to the north swell that occurs exclusively during the austral summer (November to April), whereas Haapiti is located on the western shore and is directly exposed to the prevailing southwest swell, which is of higher amplitude and occurs throughout the year.

### Sampling design

The percent cover of scleractinian corals (at genus level; 18 genera were recorded during the study period; see Supplementary Table S7), turf algae and macroalgae was recorded at 12 m depth at Tiahura, along four permanent transects of 25 m length, oriented parallel to each other and to the reef front (this station was established on a neighboring spur adjacent to the one selected for coral abundance surveys – see below). For the purpose of the present analyses, each transect was treated as a statistical replicate in all temporal contrasts. We used the Point Intercept Transect Method, with points placed every 0.25 m, to estimate cover of corals and algae. Data were collected once a year from 1991 to 2014, in March-April^11^. The significance of the interannual variability of percent cover was evaluated using the nonparametric test of Friedman, because of the absence of normality in the data set, and because these data were not independent among years. When a significant interannual variability was detected, the Wilcoxon test was used *a posteriori* to compare values of two different years.

The non-reproductive benthic phase of corals can be separated into a recruit stage, i.e. newly settled corals invisible to the naked eye on natural substrata, generally aged less than one year, and a juvenile stage, observable *in situ* (>1 cm) and typically aged at least one year^16,18^. In the present study, recruits were less than three months old; juveniles were defined as colonies with maximum diameters between 1 and 5 cm, whereas colonies >5 cm were regarded as adults^16,18^. Juvenile and adult corals were sampled along 3 replicate permanent 10 × 1 m belt-transects at each station, once a year between 2003 and 2011 and in 2014, at each of the nine stations. Transects were parallel to the depth contour and separated by ∼2 m. All the colonies at least partially enclosed in the belt transects were counted and identified to the genus level (18 and 19 genera were recorded during the study period for juvenile and adult corals, respectively; see Supplementary Table S7)^18^. Our analysis of coral cover, and abundance of juvenile and adult colonies focused on the four major coral genera (*Pocillopora, Acropora, Porites*, and *Montipora*) that together constitute >80% of the overall coral assemblages in this reef system.

To assess variation in coral settlement, 20 unglazed terracotta tiles (11 × 11 × 1 cm) were deployed at each station following the direct attachment method^38^. Two consecutive sets of tiles were deployed for three months (September to December and December to March)^25^ annually over a period of 13 years (2001–2002 to 2013–2014). This time frame was chosen because the main recruitment period in Moorea is December to March, with comparatively fewer recruits in September–December^25^. For the purpose of this survey which focused on interannual changes rather than seasonal variability, we summed the number of coral recruits over these two consecutive periods (Sept. –Dec. and Dec. –March) to estimate ‘annual’ recruitment rates. Once collected from the reefs, settlement tiles were bleached and sun-dried, and then coral recruits present on each surface of the tiles (upper-side, under-side, and edges) were counted and identified using a dissecting microscope. At this stage of development, the micro-architecture of the corallum is not sufficiently developed to allow high resolution identification, and only three families (Acroporidae, Pocilloporidae, Poritidae) of coral recruits can be reliably distinguished^39^. All other families that could not be consistently distinguished were compiled into a category of “others”. At Moorea, the family Acroporidae only encompasses three genera (*Acropora*, *Montipora*, and the uncommon *Astreopora*), whereas Pocilloporidae and Poritidae are only represented by one genus (*Pocillopora* and *Porites*, respectively).

### Statistical analyses

We used generalized linear mixed effects models (GLMMs) to evaluate if the trajectories (covariable time) of coral taxa at the different life stages varied with reef locations (Tiahura, Vaipahu and Haapiti), depths (6, 12, and 18 m) and stations (location × depth). Coral abundances were modelled using negative binomial distribution, as a number of coral colonies was recorded each year in an additive fashion.

For each coral taxa and stages, four models (see equations below) were used to fit the observed data. The model 0, called the null-model, fit the abundance of coral recruits, juveniles or adults without explanatory variables. In this case, the variability in counts of coral colonies was purely random. In Model 1, Time was added as an explanatory variable, in order to take into account the temporal variability. In Models 2 and 3, Location and Depth were added to Model 1, respectively. In Model 4, Station was added as an explanatory variable to Model 1. Random slopes and intercepts and measurements error terms were also formulated for each model. Each explanatory variable was considered as categorical with Time centered on the year 2007 and Haapiti 6 m used as an arbitrary Location and Depth reference.

The abundance (*Ab*) for each coral stages (*f*) at a station *i* and at time *j* was modelled as follows:

Model 0: no covariables (no significant spatio-temporal pattern)

Level 1: *Ab*_*fij*_ = *β*_*0i*_ + *ε*_*ij*_

Level 2: *β*_*0i*_ = *β*_*0*_ + *u*_*0i*_

Model 1: Time (temporal variability)

Level 1: *Ab*_*fij*_ = *β*_*0i*_ + *β*_*1i*_(*Time*)_*j*_ + *ε*_*ij*_

Level 2: *β*_*0i*_ = *β*_*0*_ + *u*_*0i*_

*β*_*1i*_ = *β*_*1*_ + *u*_*1i*_

Model 2: Location (different trajectories among locations)

Level 1: *Ab*_*fij*_ = *β*_*0i*_* + β*_*1i*_(*Time*)_*j*_ + *ε*_*ij*_

Level 2: *β*_*0i*_ = *β*_*0*_ + *β*_*2*_(*Location*) + *u*_*0i*_

*β1i* = *β1* + *β2*(*Location*_ij_) + *u*_*1i*_

Model 3: Depth (different trajectories among depths)

Level 1: *Ab*_*fij*_ = *β*_*0i*_ + *β*_*1i*_(*Time*)_*j*_ + *ε*_*ij*_

Level 2: *β*_*0i*_ = *β*_*0*_ + *β*_*2*_(*Depth*)_*ij*_ + *u*_*0i*_

*β*_*1i*_ = *β*_*1*_ + *β*_2_(*Depth*)_*ij*_ + *u*_*1i*_

Model 4: Station (different trajectories among stations, i.e. per depths within locations)

Level 1: *Ab*_*fij*_ = *β*_*0i*_ + *β*_*1i*_(*Time*)_*j*_ + *ε*_*ij*_

Level 2: *β*_*0i*_ = *β*_*0*_ + *β*_*2*_(*Location*)_*ij*_ + *β*_*3*_(*Depth*(*Location*))_*ij*_ + *u*_*0i*_

*β*_1*i*_ = *β*_*1*_ + *β*_*2*_(*Location*)_*ij*_ + *β*_*3*_(*Depth*(*Location*))_*ij*_ + *u*_*1i*_

The best model formulations were identified using Akaike Information Criterion (AIC) and Chi² deviance tests^40^. Model validity was controlled by plotting the estimated data *vs*. observed data and model residuals. All statistics were performed in R version 3.1.0^41^ complemented by the package glmmADMB^42^.

Model outputs were then used to determine coral recovery using pairwise comparisons where the first year of survey was considered as reference. Post-hoc analyses were conducted at the level of stations, depths (all locations pooled), locations (all depths pooled), and for all stations pooled. The multcomp package^43^ was used to perform these tests.

Finally, we used non-metric multidimensional scaling (nMDS) to examine interannual variation in the composition of the coral assemblage based on the Bray–Curtis dissimilarity index of the cover of coral genera recorded annually. This analysis was encoded in R version 3.1.0^41^.

### Data availability

Data used in this paper can be accessed at http://www.criobe.pf.

## Electronic supplementary material

Supplementary Info
