# Supplementary material for: Recovery of coral assemblages despite acute and recurrent disturbances on a South Central Pacific reef
Source: Sci Rep. 2018 Jun 26;8:9680. doi: 10.1038/s41598-018-27891-3 (PMC6018695; doi:10.1038/s41598-018-27891-3)
Supplement: Supplementary file 1 — Supplementary Info [file 41598_2018_27891_MOESM1_ESM.pdf]

Recovery of coral assemblages despite acute and recurrent disturbances on a South Central Pacific reef

Mehdi Adjeroud, Mohsen Kayal, Claudie Iborra-Cantonnet, Julie Vercelloni, Pauline Bosserelle, Vetea Liao, Yannick Chancerelle, Joachim Claudet, Lucie Penin

Supplementary Information

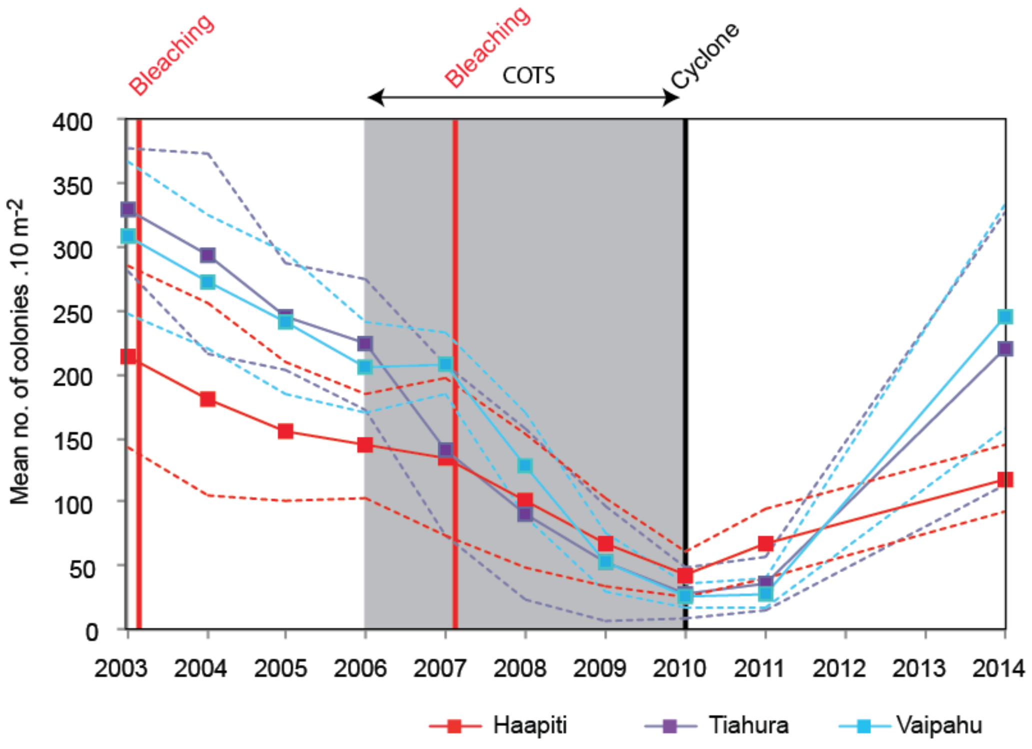

Figure S1. Temporal dynamics in adult coral abundance (all 19 genera pooled) at the three locations (all three depths pooled at each location) impacted by the four disturbances between 2003 and 2014. Dotted lines represent standard deviation.

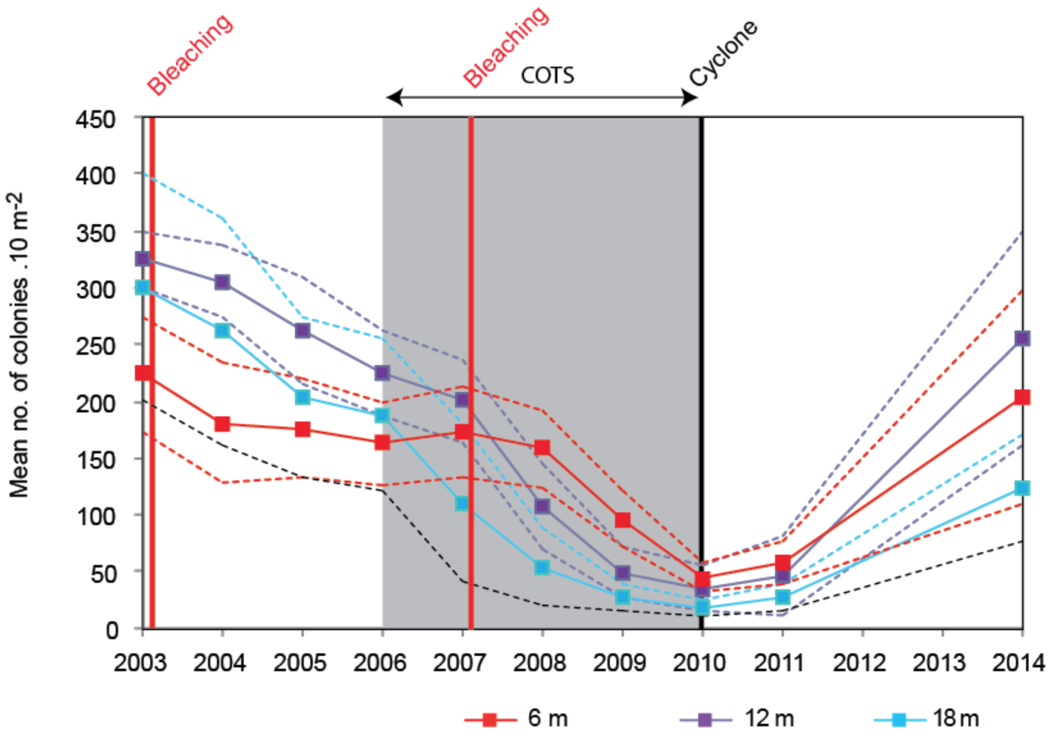

Figure S2. Temporal dynamics in adult coral abundance (all 19 genera pooled) at the three depths (all three locations pooled for each depth) impacted by the four disturbances between 2003 and 2014. Dotted lines represent standard deviation.

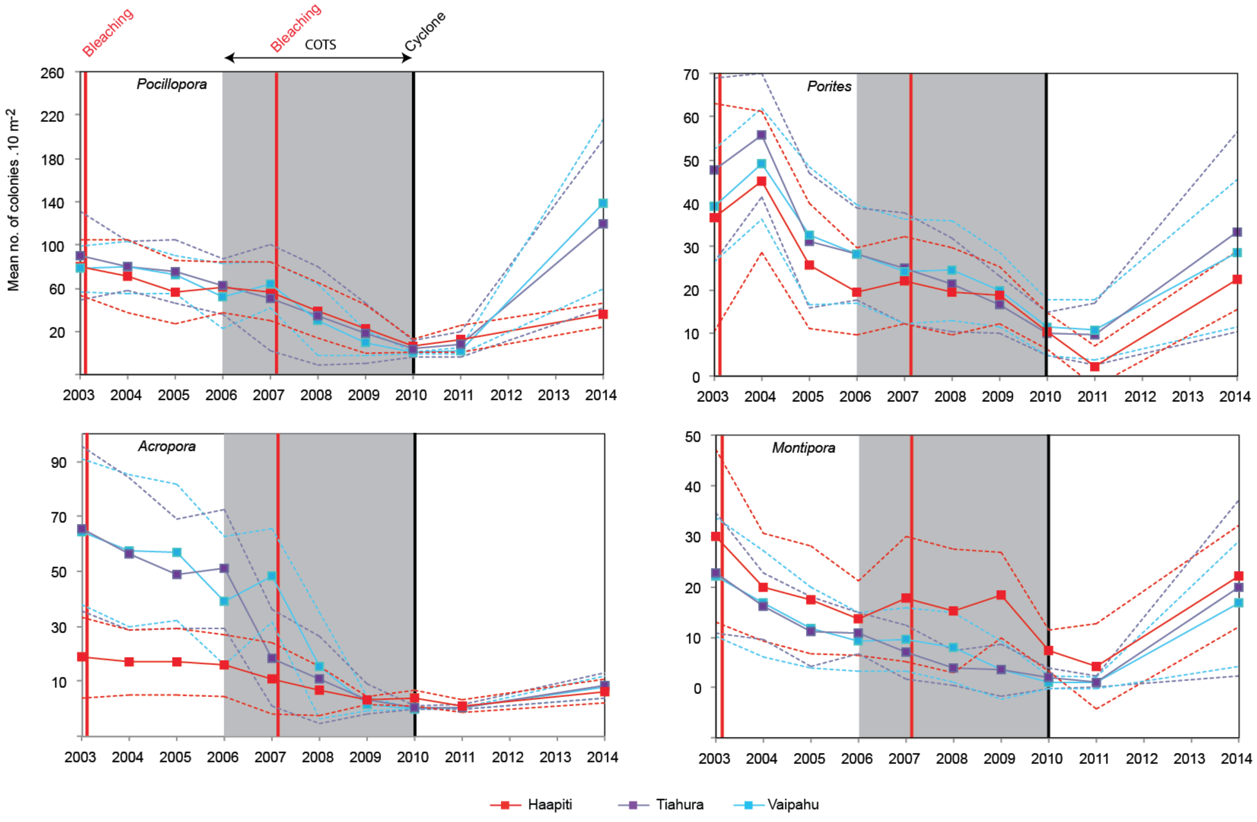

Figure S3. Temporal dynamics in adult coral abundance of the four dominant genera at the three locations (all three depths pooled at each location) impacted by the four disturbances between 2003 and 2014. Dotted lines represent standard deviation.

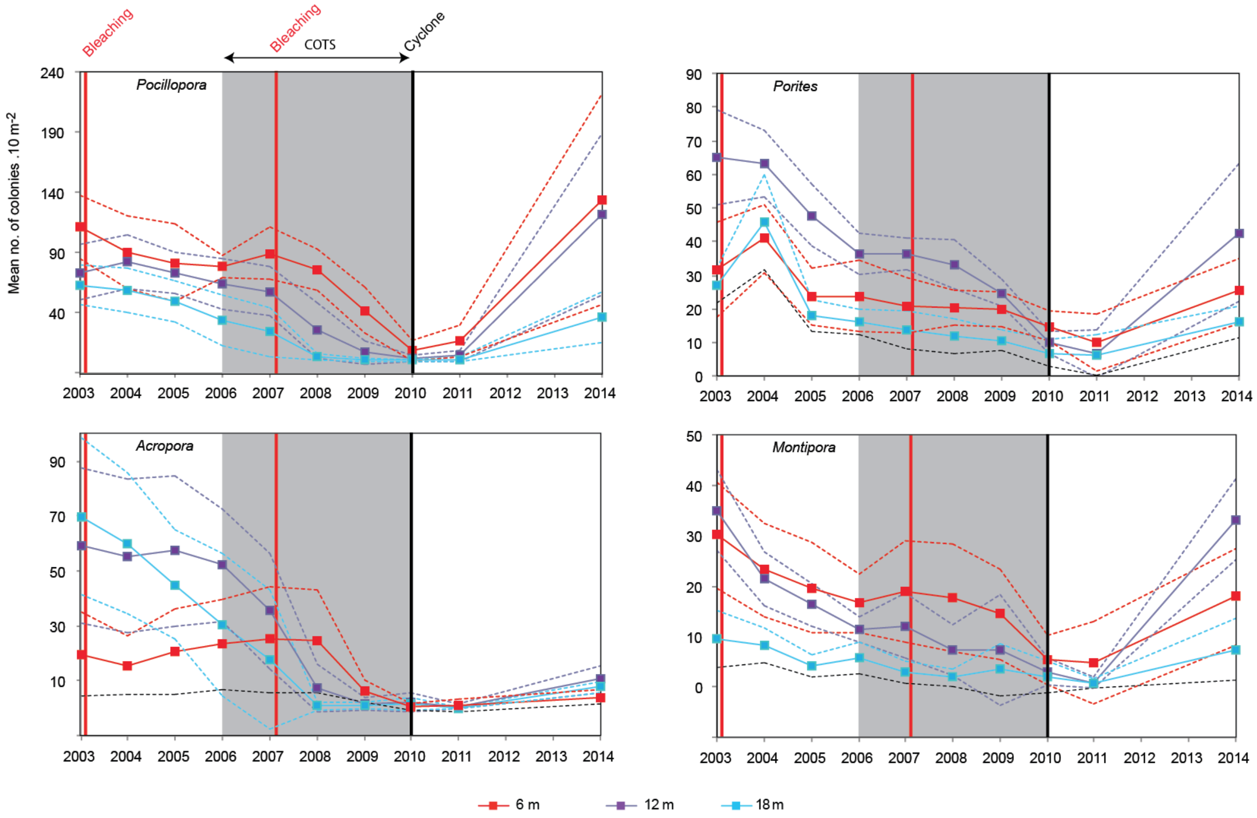

Figure S4. Temporal dynamics in adult coral abundance of the four dominant genera at the three depths (all three locations pooled for each depth) impacted by the four disturbances between 2003 and 2014. Dotted lines represent standard deviation.

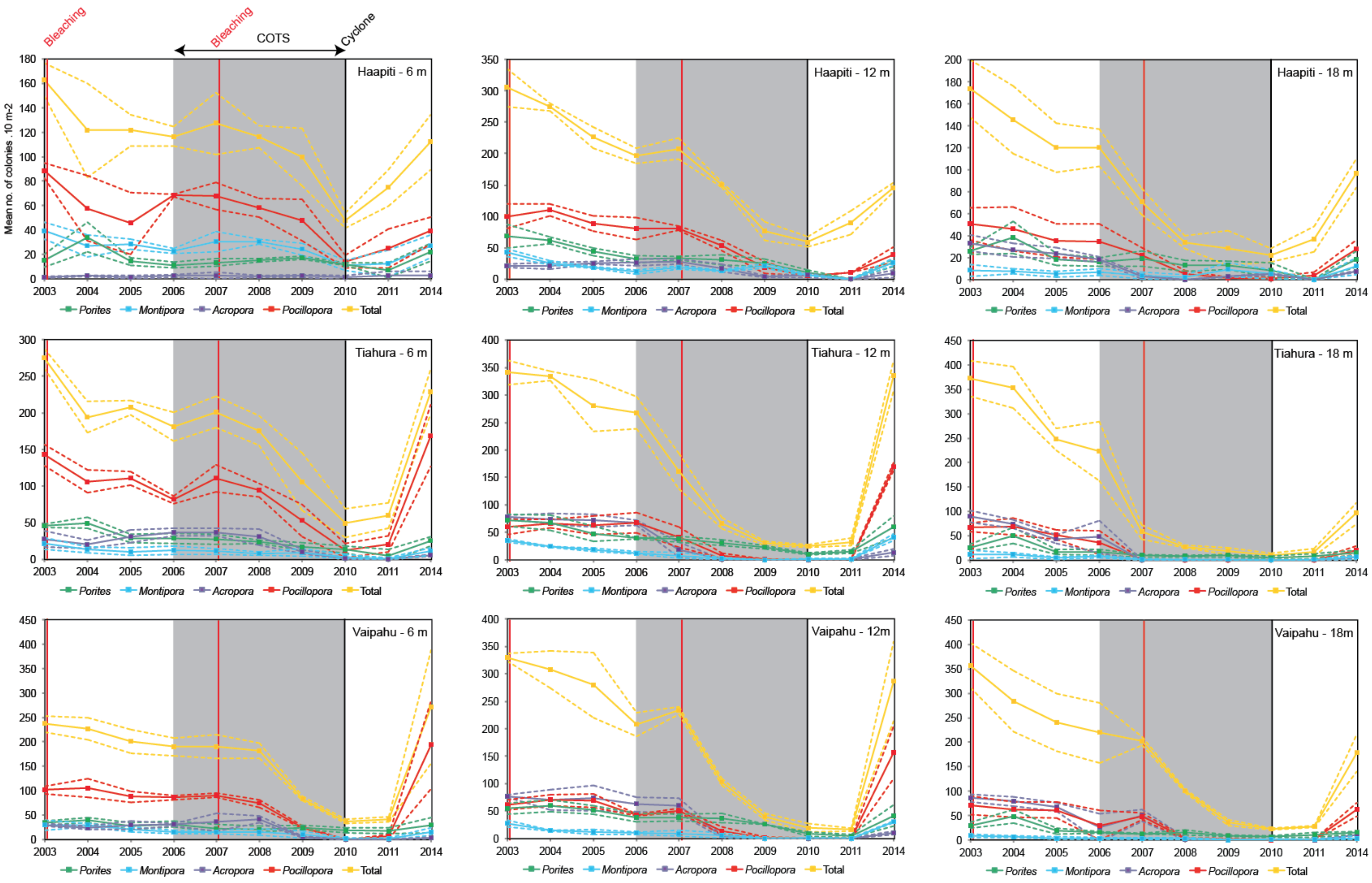

Figure S5. Temporal dynamics in adult coral abundance of the four dominant genera at the nine stations impacted by the four disturbances between 2003 and 2014. Dotted lines represent standard deviation.

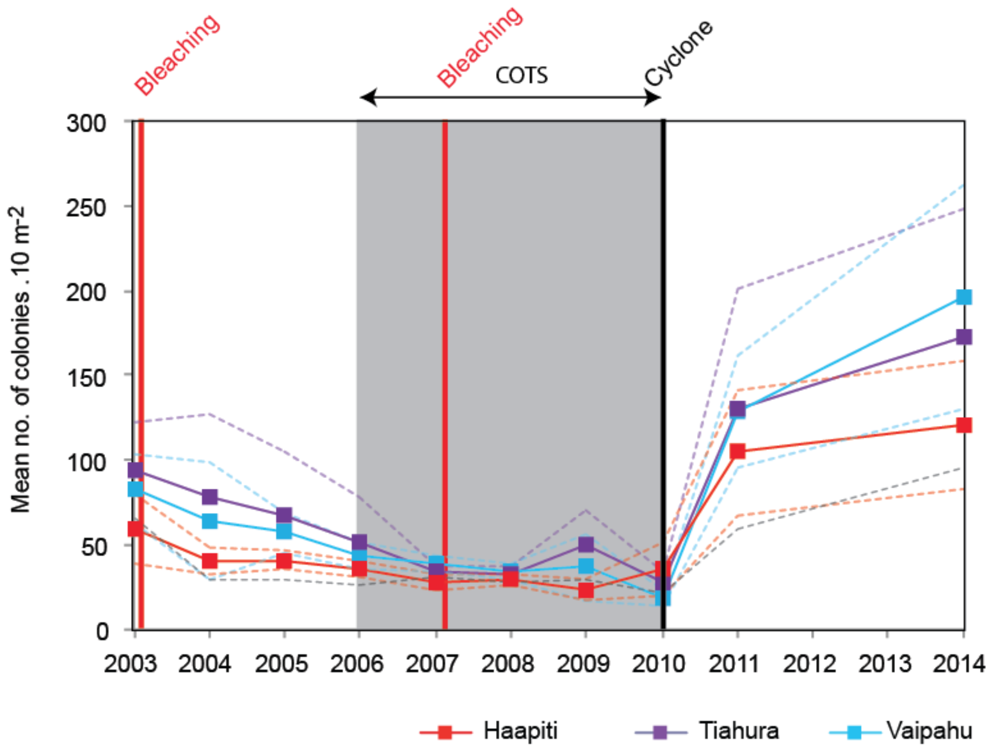

Figure S6. Temporal dynamics in juvenile coral abundance (all 19 genera pooled) at the three locations (all three depths pooled at each location) impacted by the four disturbances between 2003 and 2014. Dotted lines represent standard deviation.

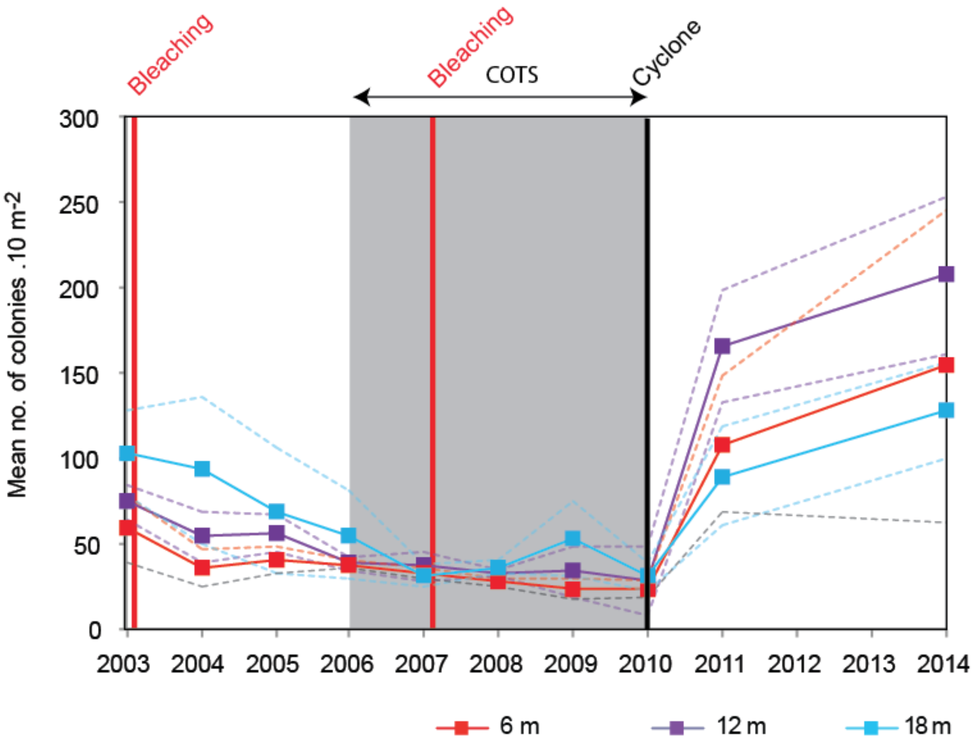

Figure S7. Temporal dynamics in juvenile coral abundance (all 19 genera pooled) at the three depths (all three locations pooled for each depth) impacted by the four disturbances between 2003 and 2014. Dotted lines represent standard deviation.

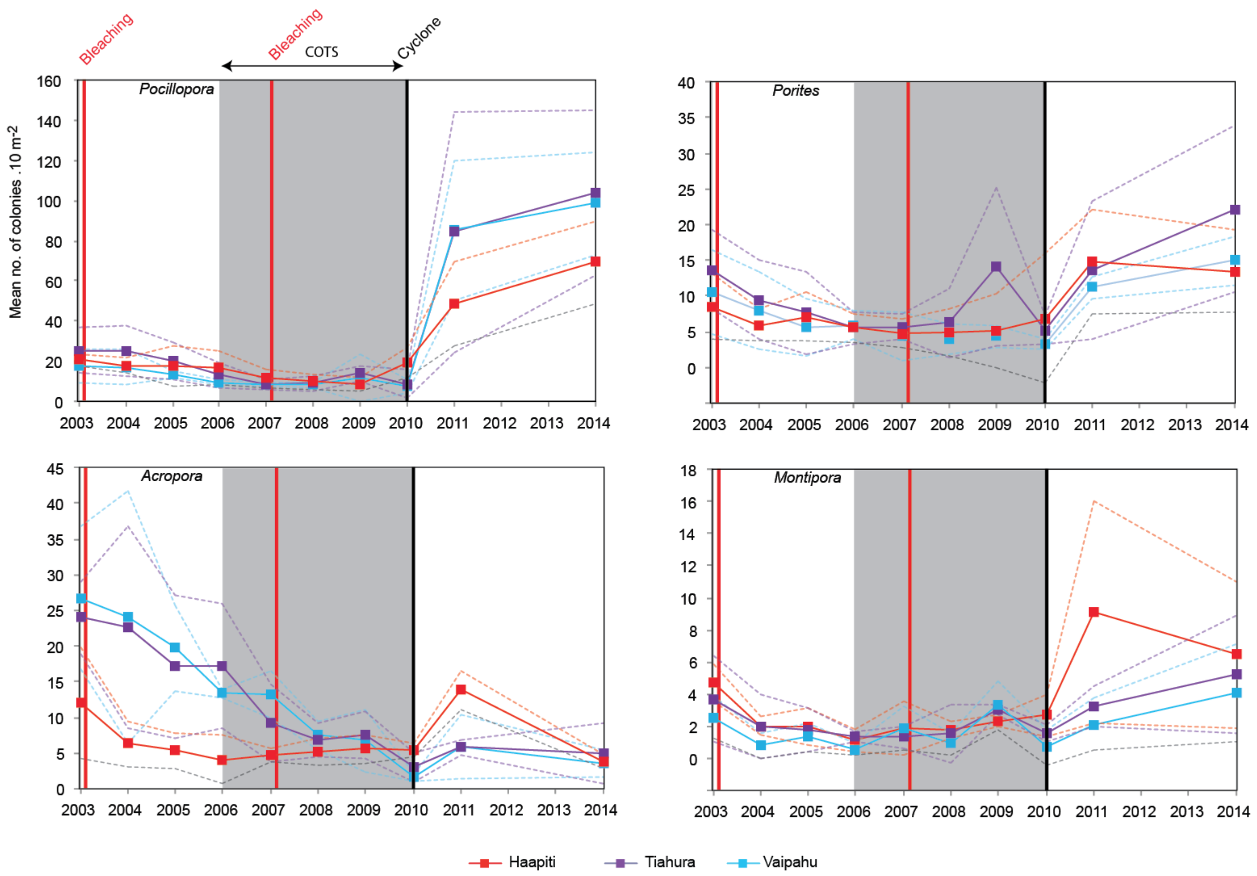

Figure S8. Temporal dynamics in juvenile coral abundance of the four dominant genera at the three locations (all three depths pooled at each location) impacted by the four disturbances between 2003 and 2014. Dotted lines represent standard deviation.

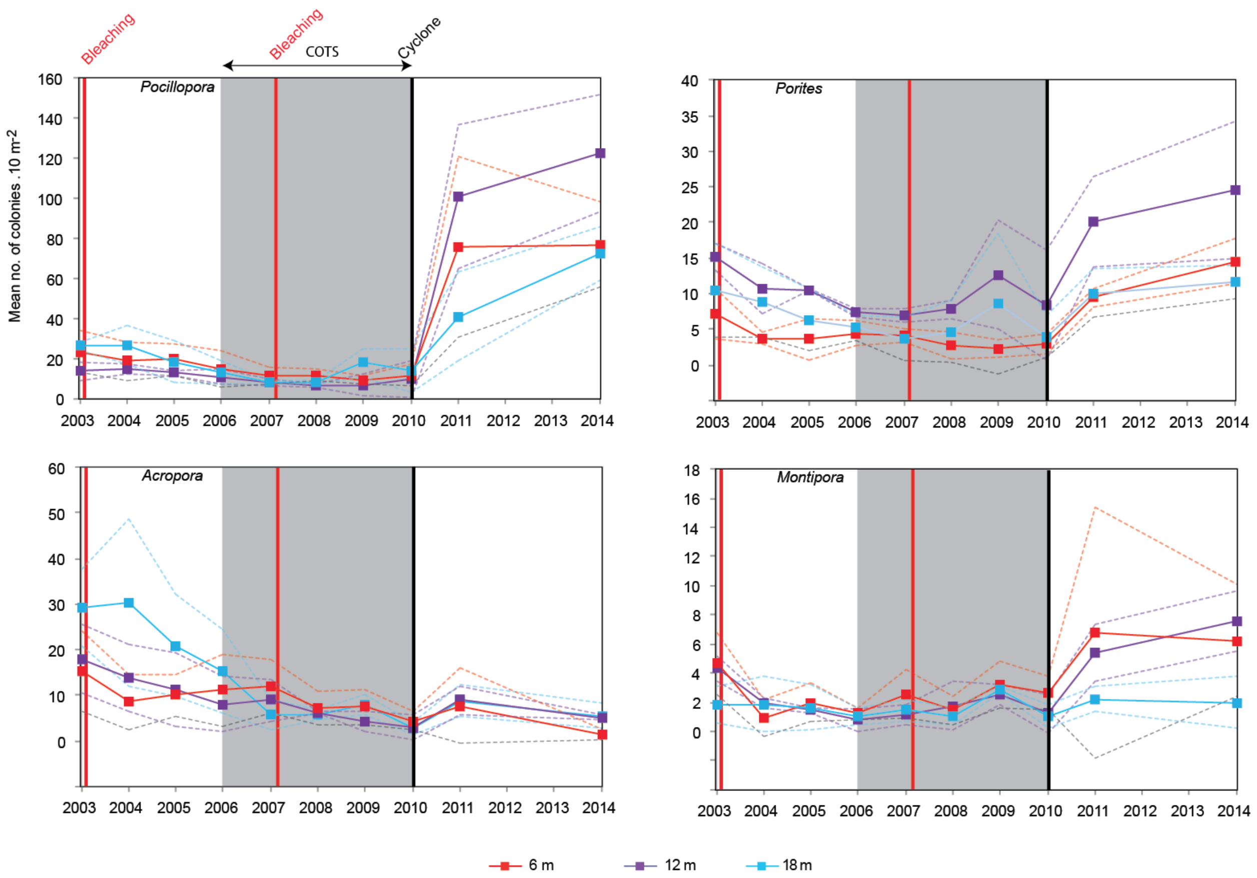

Figure S9. Temporal dynamics in juvenile coral abundance of the four dominant genera at the three depths (all three locations pooled for each depth) impacted by the four disturbances between 2003 and 2014. Dotted lines represent standard deviation.

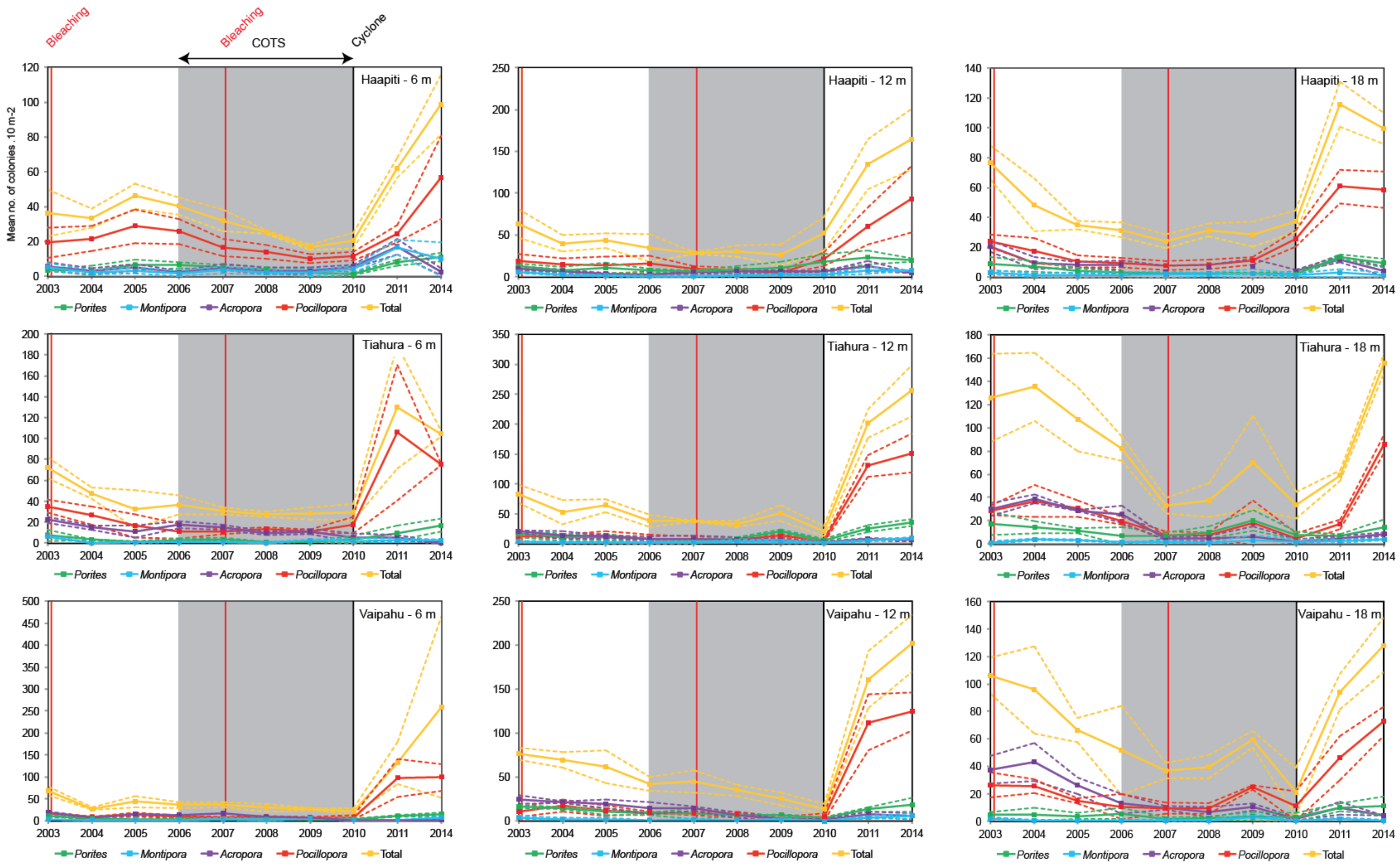

Figure S10. Temporal dynamics in juvenile coral abundance of the four dominant genera at the nine stations impacted by the four disturbances between 2003 and 2014. Dotted lines represent standard deviation.

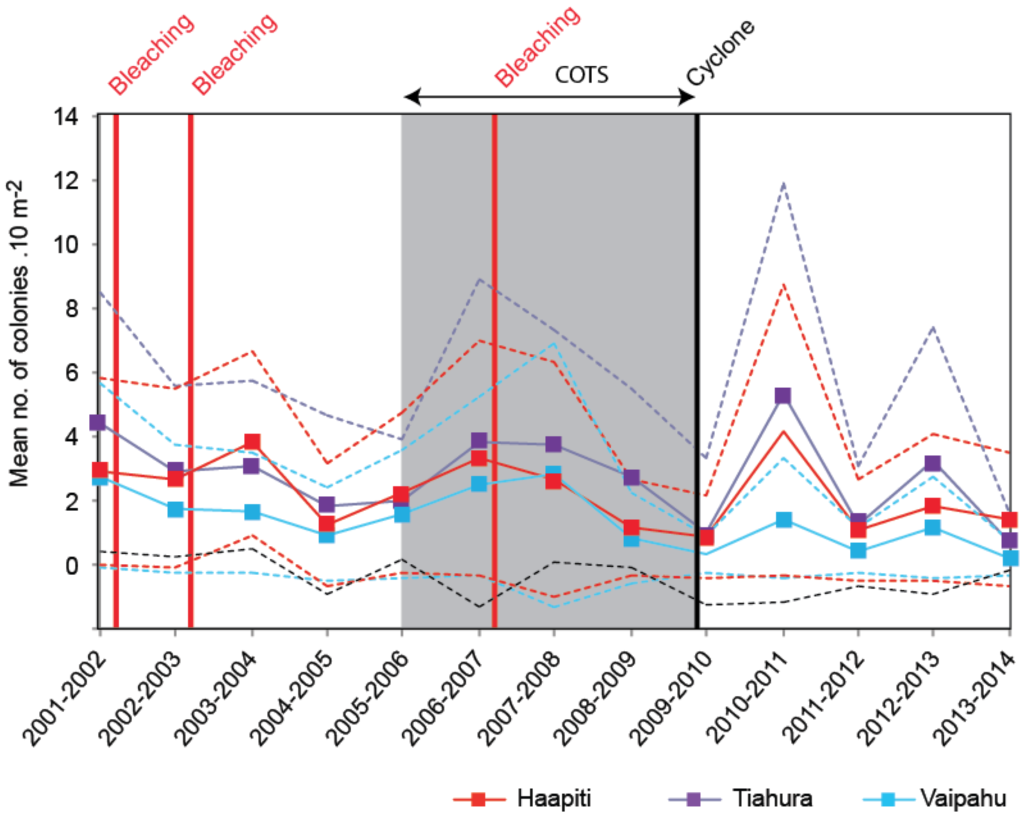

Figure S11. Temporal dynamics in coral recruit abundance at the three locations (all three depths pooled at each location) impacted by the four disturbances between Sept. 2001 – March 2002 and Sept. 2013 – March 2014. Dotted lines represent standard deviation.

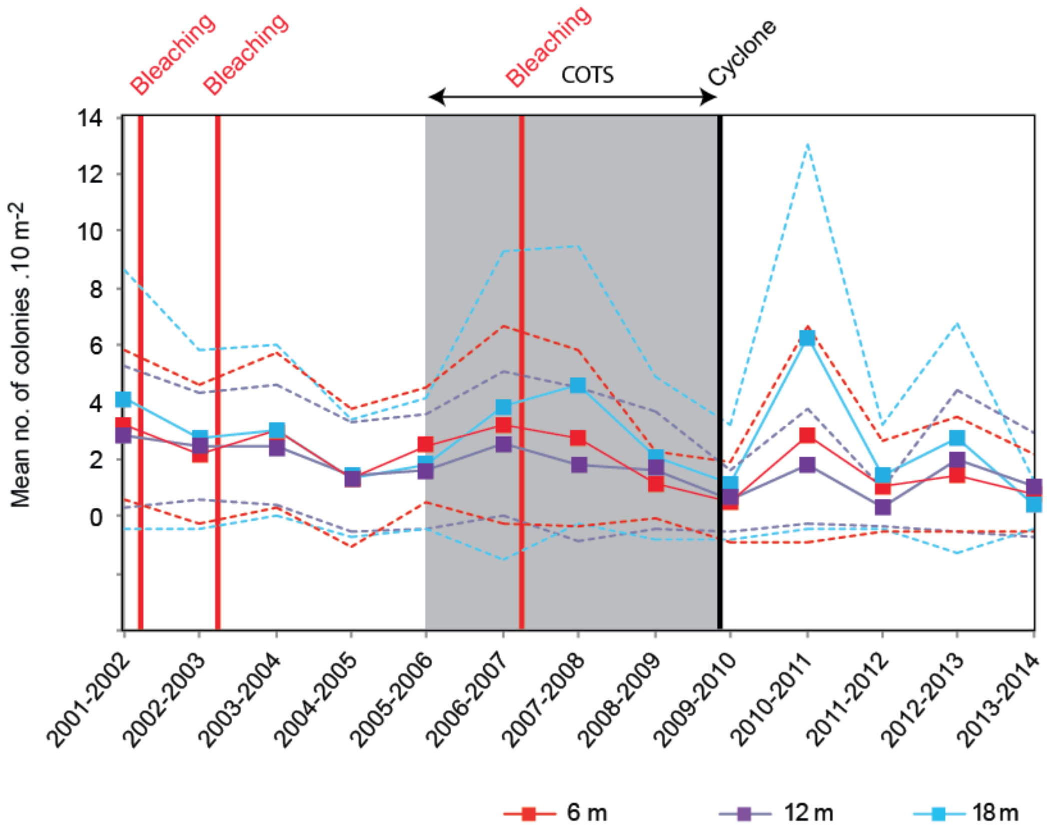

Figure S12. Temporal dynamics in coral recruit abundance at the three depths (all three locations pooled for each depth) impacted by the four disturbances between Sept. 2001 – March 2002 and Sept. 2013 – March 2014. Dotted lines represent standard deviation.

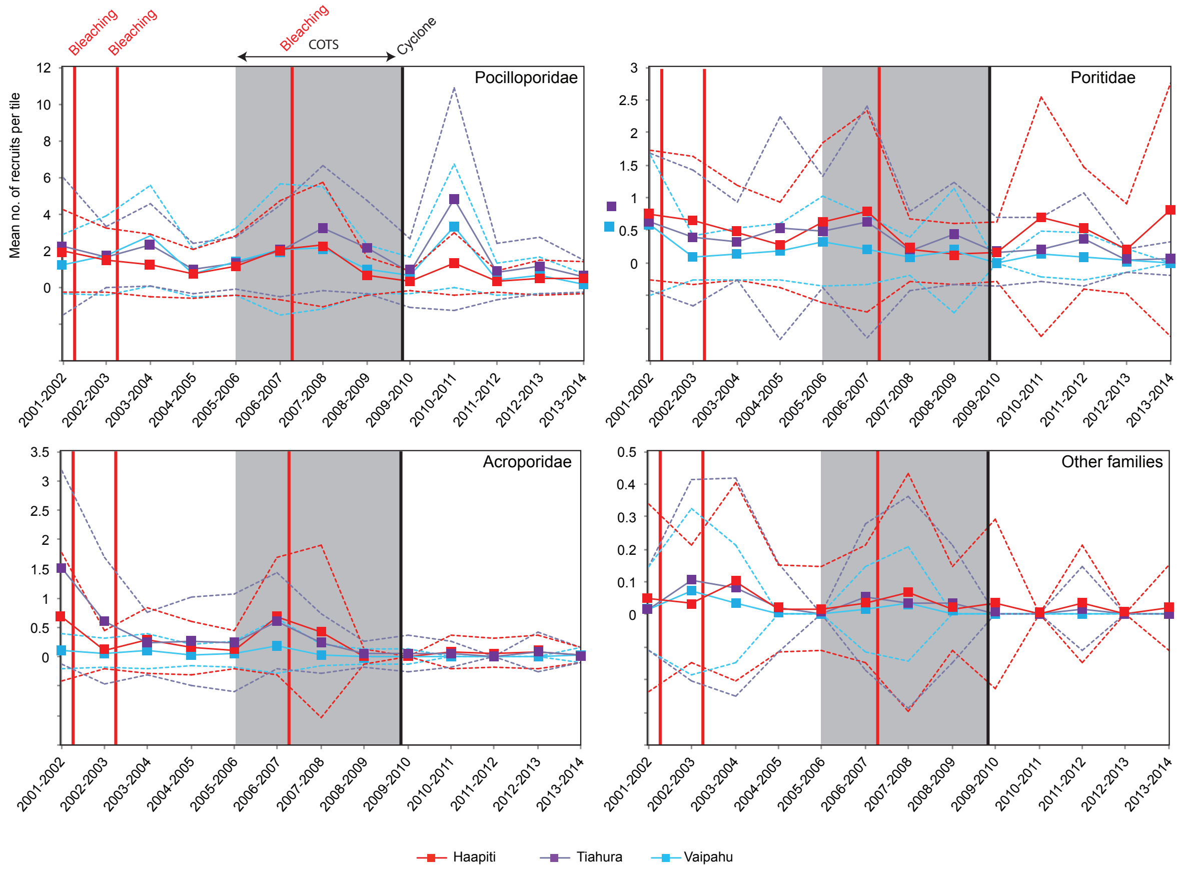

Figure S13. Temporal dynamics in the abundance of the four major categories of coral recruits at the three locations (all three depths pooled at each location) impacted by the four disturbances between Sept. 2001 – March 2002 and Sept. 2013 – March 2014. Dotted lines represent standard deviation.

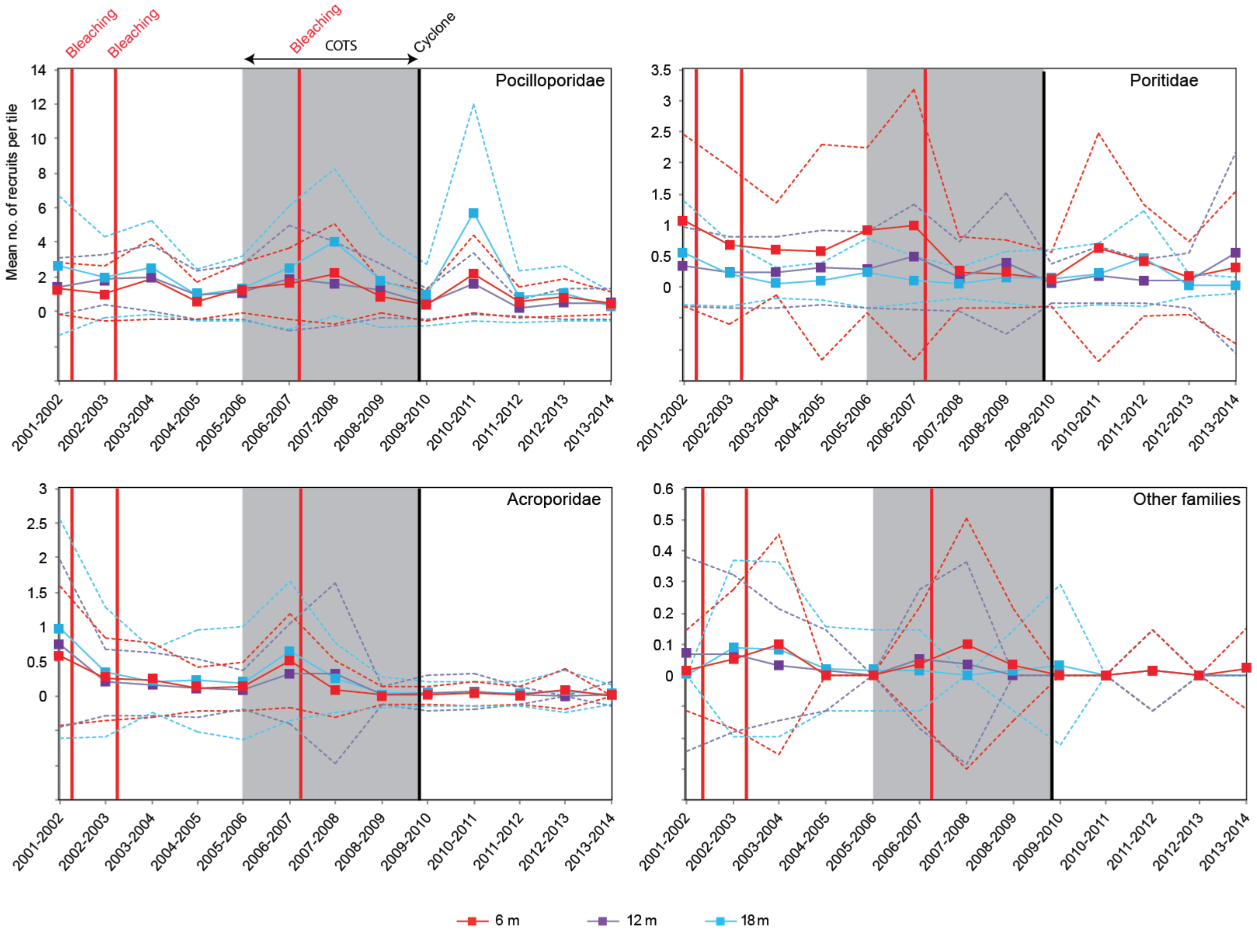

Figure S14. Temporal dynamics in the abundance of the four major categories of coral recruits at the three depths (all three locations pooled for each depth) impacted by the four disturbances between Sept. 2001 – March 2002 and Sept. 2013 – March 2014. Dotted lines represent standard deviation.

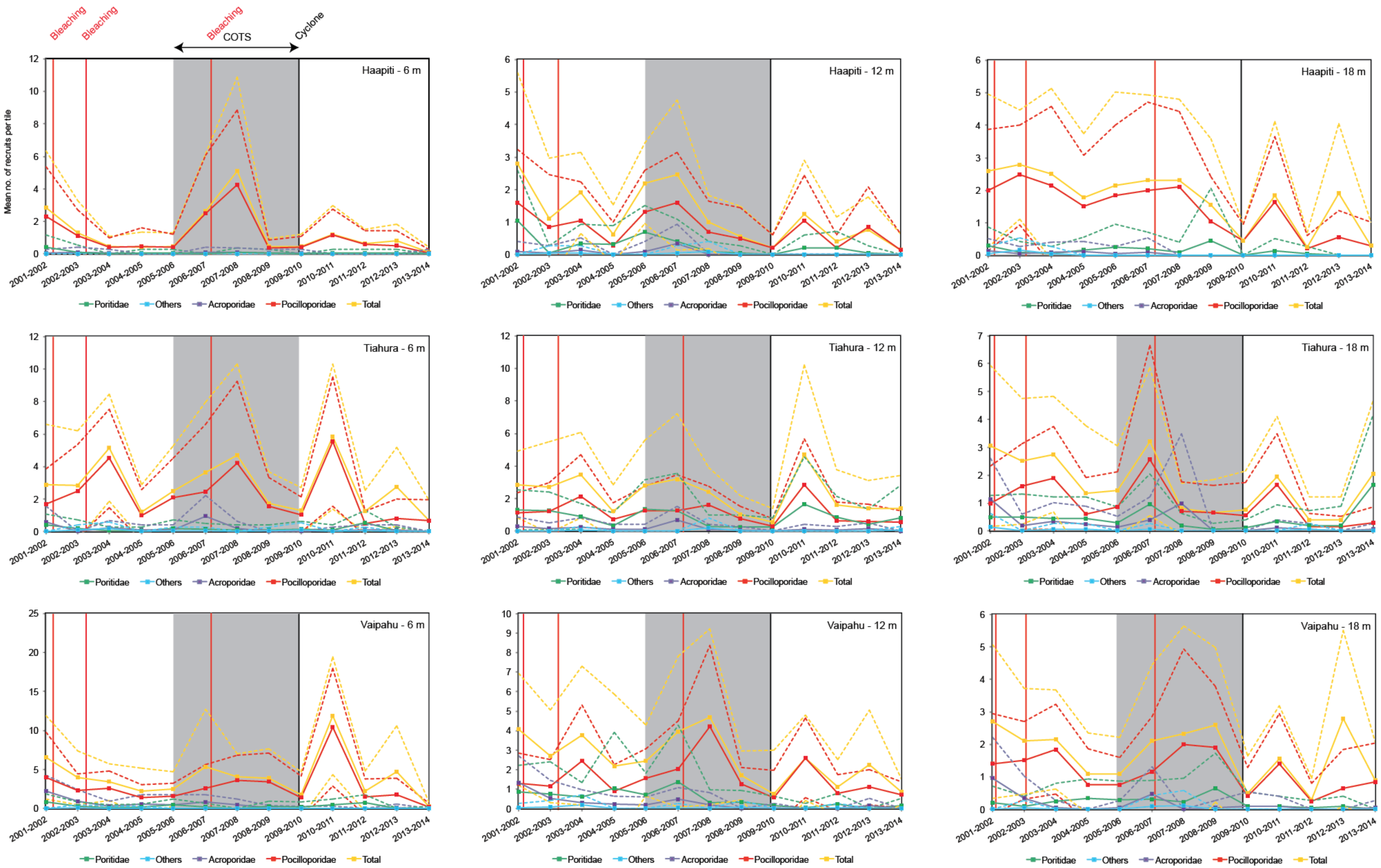

Figure S15. Temporal dynamics in the abundance of the four major categories of coral recruits at the nine stations impacted by the four disturbances between Sept. 2001 – March 2002 and Sept. 2013 – March 2014. Dotted lines represent standard deviation.

Table S1. Differences between pairs of years for coral cover (all 18 genera pooled). Significant differences are indicated in red (Wilcoxon tests, p-values < 5%). The interannual variability in cover among years was significant (Friedman test, p < 0.001).

	1991	1992	1993	1994	1995	1996	1997	1998	1999	2000	2001	2002	2003	2004	2005	2006	2007	2008	2009	2010	2011	2012	2013
**1992**	0.057																						
**1993**	0.029	0.886																					
**1994**	0.029	0.686	0.486																				
**1995**	0.029	0.686	0.343	0.661																			
**1996**	0.029	0.663	0.309	0.468	0.663																		
**1997**	0.081	0.200	0.029	0.057	0.146	0.144																	
**1998**	0.200	0.114	0.029	0.042	0.108	0.110	0.561																
**1999**	0.191	0.110	0.029	0.055	0.183	0.108	0.663	0.882															
**2000**	0.309	0.191	0.029	0.029	0.029	0.028	0.191	1.000	0.884														
**2001**	0.770	0.041	0.029	0.029	0.029	0.028	0.059	0.191	0.297	0.058													
**2002**	0.663	0.029	0.029	0.029	0.029	0.028	0.029	0.663	0.557	0.108	0.661												
**2003**	0.200	0.057	0.029	0.029	0.029	0.029	0.114	1.000	0.663	0.559	0.191	0.309											
**2004**	0.886	0.029	0.029	0.029	0.029	0.029	0.029	0.561	0.381	0.110	0.885	1.000	0.465										
**2005**	0.657	0.041	0.029	0.029	0.029	0.028	0.059	0.309	0.378	0.058	0.760	1.000	0.191	0.885									
**2006**	0.343	0.029	0.029	0.029	0.029	0.029	0.042	1.000	0.663	0.144	0.309	0.309	1.000	0.465	0.309								
**2007**	0.029	1.000	1.000	0.110	0.041	0.028	0.029	0.029	0.028	0.028	0.028	0.028	0.029	0.029	0.028	0.029							
**2008**	0.029	0.029	0.029	0.029	0.029	0.029	0.029	0.029	0.029	0.029	0.029	0.029	0.029	0.029	0.029	0.029	0.029						
**2009**	0.029	0.029	0.029	0.029	0.029	0.028	0.029	0.029	0.028	0.028	0.028	0.028	0.029	0.029	0.028	0.029	0.028	0.144					
**2010**	0.028	0.028	0.028	0.028	0.028	0.027	0.028	0.028	0.027	0.027	0.027	0.027	0.028	0.028	0.027	0.028	0.027	0.028	0.089				
**2011**	0.027	0.027	0.027	0.027	0.027	0.026	0.027	0.027	0.026	0.026	0.026	0.026	0.027	0.027	0.026	0.027	0.026	0.027	0.137	0.608			
**2012**	0.029	0.029	0.029	0.029	0.029	0.029	0.029	0.029	0.029	0.029	0.029	0.029	0.029	0.029	0.029	0.029	0.029	0.146	0.653	0.053	0.065		
**2013**	0.029	0.029	0.029	0.029	0.029	0.028	0.029	0.029	0.028	0.028	0.028	0.028	0.029	0.029	0.028	0.029	0.028	0.770	0.297	0.052	0.063	0.381	
**2014**	0.029	0.686	0.686	0.057	0.057	0.029	0.029	0.029	0.029	0.029	0.029	0.029	0.029	0.029	0.029	0.029	0.309	0.057	0.029	0.028	0.027	0.029	0.055

Table S2. Differences between pairs of years for cover of turf algae. Significant differences are indicated in red (Wilcoxon tests, p-values < 5%).

The interannual variability in cover among years was significant (Friedman test, $p < 0.001$).

	1991	1992	1993	1994	1995	1996	1997	1998	1999	2000	2001	2002	2003	2004	2005	2006	2007	2008	2009	2010	2011	2012	2013
**1992**	0.029																						
**1993**	0.029	0.080																					
**1994**	0.029	0.028	0.029																				
**1995**	0.029	0.465	0.770	0.028																			
**1996**	0.029	0.080	0.661	0.104	0.381																		
**1997**	0.029	0.770	0.306	0.029	0.663	0.245																	
**1998**	0.029	0.055	0.029	0.029	0.309	0.029	0.245																
**1999**	0.029	0.770	0.146	0.029	0.381	0.114	0.772	0.772															
**2000**	0.343	0.029	0.029	0.029	0.110	0.029	0.114	0.245	0.245														
**2001**	0.146	0.144	0.029	0.029	0.110	0.029	0.146	0.384	0.245	0.886													
**2002**	0.146	0.029	0.029	0.029	0.110	0.029	0.081	0.146	0.200	1.000	0.884												
**2003**	0.183	0.028	0.029	0.028	0.028	0.029	0.029	0.029	0.029	1.000	0.663	0.770											
**2004**	0.104	0.028	0.029	0.028	0.058	0.029	0.059	0.110	0.110	0.770	1.000	1.000	0.442										
**2005**	0.663	0.028	0.029	0.028	0.028	0.029	0.029	0.029	0.029	0.663	0.191	0.191	0.189	0.078									
**2006**	0.561	0.029	0.029	0.029	0.029	0.029	0.029	0.029	0.029	0.686	0.189	0.245	0.191	0.059	1.000								
**2007**	0.029	0.183	0.561	0.243	0.468	0.886	0.245	0.042	0.146	0.029	0.057	0.029	0.029	0.029	0.029	0.029							
**2008**	0.029	0.029	0.029	0.309	0.029	0.081	0.029	0.029	0.029	0.029	0.029	0.029	0.029	0.029	0.029	0.029	0.114						
**2009**	0.029	0.059	0.245	1.000	0.110	0.486	0.114	0.029	0.057	0.029	0.029	0.029	0.029	0.029	0.029	0.029	0.486	0.306					
**2010**	0.029	0.029	0.081	0.885	0.110	0.245	0.042	0.029	0.042	0.029	0.029	0.029	0.029	0.029	0.029	0.029	0.465	0.384	0.886				
**2011**	0.029	0.028	0.059	0.306	0.028	0.110	0.029	0.029	0.029	0.029	0.029	0.029	0.028	0.028	0.028	0.029	0.110	0.243	1.000	0.663			
**2012**	0.029	0.028	0.110	1.000	0.091	0.309	0.029	0.029	0.029	0.029	0.029	0.029	0.028	0.028	0.028	0.029	0.309	0.663	0.663	0.663	1.000		
**2013**	0.029	0.309	0.686	0.059	0.301	0.886	0.200	0.081	0.114	0.029	0.057	0.029	0.029	0.029	0.029	0.029	0.886	0.057	0.343	0.343	0.080	0.460	
**2014**	0.029	0.080	0.343	0.381	0.191	0.686	0.114	0.029	0.081	0.029	0.042	0.029	0.029	0.029	0.029	0.029	0.772	0.200	0.686	0.686	0.191	0.663	0.384

Table S3. Differences between pairs of years for cover of macroalgae. Significant differences are indicated in red (Wilcoxon tests, p-values < 5%).

The interannual variability in cover among years was significant (Friedman test, $p < 0.001$).

	**1991**	**1992**	**1993**	**1994**	**1995**	**1996**	**1997**	**1998**	**1999**	**2000**	**2001**	**2002**	**2003**	**2004**	**2005**	**2006**	**2007**	**2008**	**2009**	**2010**	**2011**	**2012**	**2013**
**1992**	0.453																						
**1993**	1.000	0.453																					
**1994**	1.000	0.453	1.000																				
**1995**	0.620	0.186	0.505	0.620																			
**1996**	1.000	0.453	1.000	1.000	0.620																		
**1997**	0.453	-------	0.453	0.453	0.186	0.453																	
**1998**	0.453	-------	0.453	0.453	0.186	0.453	-------																
**1999**	0.453	-------	0.453	0.453	0.186	0.453	-------	-------															
**2000**	0.453	-------	0.453	0.453	0.186	0.453	-------	-------	-------														
**2001**	0.453	-------	0.453	0.453	0.186	0.453	-------	-------	-------	-------													
**2002**	0.739	0.186	0.505	0.739	1.000	0.739	0.186	0.186	0.186	0.186	0.186												
**2003**	1.000	0.453	1.000	1.000	0.620	1.000	0.453	0.453	0.453	0.453	0.453	0.739											
**2004**	0.278	0.069	0.163	0.278	0.766	0.278	0.069	0.069	0.069	0.069	0.069	0.454	0.278										
**2005**	0.868	0.181	0.608	0.868	0.874	0.868	0.181	0.181	0.181	0.181	0.181	0.874	0.868	0.288									
**2006**	0.739	0.186	0.505	0.739	1.000	0.739	0.186	0.186	0.186	0.186	0.186	1.000	0.739	0.454	0.874								
**2007**	0.868	0.181	0.608	0.868	0.874	0.868	0.181	0.181	0.181	0.181	0.181	0.874	0.868	0.288	1.000	0.874							
**2008**	0.074	0.021	0.037	0.074	0.186	0.074	0.021	0.021	0.021	0.021	0.021	0.106	0.074	0.306	0.053	0.106	0.053						
**2009**	0.620	0.186	0.505	0.620	1.000	0.620	0.186	0.186	0.186	0.186	0.186	1.000	0.620	0.653	0.874	1.000	0.874	0.144					
**2010**	0.453	-------	0.453	0.453	0.186	0.453	-------	-------	-------	-------	-------	0.186	0.453	0.069	0.181	0.186	0.181	0.021	0.186				
**2011**	0.036	0.020	0.026	0.036	0.240	0.036	0.020	0.020	0.020	0.020	0.020	0.040	0.036	0.137	0.027	0.040	0.027	1.000	0.102	0.020			
**2012**	0.206	0.067	0.122	0.206	0.457	0.206	0.067	0.067	0.067	0.067	0.067	0.288	0.206	0.766	0.178	0.288	0.178	0.657	0.457	0.067	0.457		
**2013**	0.027	0.021	0.027	0.027	0.041	0.027	0.021	0.021	0.021	0.021	0.021	0.029	0.027	0.029	0.028	0.029	0.028	0.108	0.029	0.021	0.041	0.059	
**2014**	0.026	0.020	0.026	0.026	0.058	0.026	0.020	0.020	0.020	0.020	0.020	0.028	0.026	0.041	0.027	0.028	0.027	0.309	0.040	0.020	0.102	0.102	0.381

Table S4. Deviance table of the generalized linear mixed-effect models (GLMMs) best describing the dynamics of coral stages and taxa. Four candidate models with different levels of complexity were tested, and the best model formulation was identified using Akaike Information Criterion (AIC) and Chi² deviance tests.

Life stage	Taxon	Best model	Covariable	p-value
Adults	*Pocillopora*	Model 3	Time	0.025
			Depth	< 0.001
			Time × Depth	0.002
	Porites	Model 3	Time	0.002
			Depth	< 0.001
			Time × Depth	0.027
	Acropora	Model 2	Time	0.002
			Location	0.002
			Time × Location	0.065
	Montipora	Model 3	Time	0.014
			Depth	< 0.001
			Time × Depth	0.731
	All 19 genera	Model 3	Time	0.041
			Depth	0.145
			Time × Depth	0.053
Juveniles	*Pocillopora*	Model 1	Time	< 0.001
	Porites	Model 3	Time	0.020
			Depth	< 0.001
			Time × Depth	0.328
	Acropora	Model 2	Time	0.823
			Location	0.001
			Time × Location	< 0.001
	Montipora	Model 1	Time	0.001
	All 19 genera	Model 3	Time	0.002
			Depth	0.0292
			Time × Depth	0.098
Recruits	Pocilloporidae	Model 0	-	-
	Poritidae	Model 0	-	-
	Acroporidae	Model 1	Time	0.001
	Other families	Model 0	-	-
	All taxa	Model 0	-	-

Table S5. Parameter estimates of the generalized linear mixed-effect models (GLMMs) best describing the dynamics of coral stages and taxa. Four candidate models with different levels of complexity were tested, and the best model formulation was identified using Akaike Information Criterion (AIC) and Chi² deviance tests. SE indicates standard error.

Life stage	Taxon	Best model	Level	Intercept	SE intercept	Slope	SE slope
Adults	*Pocillopora*	Model 3	Depth = 6m	4.269	0.117	-0.115	0.039
			Depth = 12m	3.625	0.182	-0.234	0.061
			Depth = 18m	3.087	0.204	-0.298	0.068
	Porites	Model 3	Depth = 6m	3.108	0.080	-0.081	0.028
			Depth = 12m	3.527	0.115	-0.127	0.038
			Depth = 18m	2.751	0.121	-0.148	0.040
	Acropora	Model 2	Site = Haapiti	2.125	0.209	-0.214	0.071
			Site = Tiahura	2.754	0.293	-0.369	0.100
			Site = Vaipahu	2.868	0.293	-0.389	0.102
	Montipora	Model 3	Depth = 6m	2.733	0.189	-0.116	0.046
			Depth = 12m	2.655	0.195	-0.069	0.060
			Depth = 18m	1.456	0.209	-0.109	0.067
	All 19 genera	Model 3	Depth = 6m	4.968	0.128	-0.071	0.038
			Depth = 12m	4.804	0.163	-0.117	0.052
			Depth = 18m	4.688	0.163	-0.189	0.052
Juveniles	*Pocillopora*	Model 1	all locations	3.173	0.082	0.153	0.027
	Porites	Model 3	Depth = 6m	1.669	0.148	0.088	0.036
			Depth = 12m	2.498	0.198	0.062	0.047
			Depth = 18m	1.954	0.200	0.019	0.049
	Acropora	Model 2	Site = Haapiti	1.883	0.118	-0.01	0.034
			Site = Tiahura	2.276	0.163	-0.212	0.047
			Site = Vaipahu	2.255	0.164	-0.247	0.047
	Montipora	Model 1	all populations	0.843	0.144	0.088	0.027
	All 19 genera	Model 3	Depth = 6m	3.932	0.124	0.100	0.032
			Depth = 12m	4.214	0.152	0.109	0.044
			Depth = 18m	4.225	0.151	0.003	0.045
Recruits	Pocilloporidae	Model 0	all locations	0.384	0.138	-	-
	Poritidae	Model 0	all locations	-1.186	0.232	-	-
	Acroporidae	Model 1	all locations	-2.053	0.443	-0.242	0.075
	Other families	Model 0	all locations	-3.649	0.597	-	-
	All taxa	Model 0	all locations	0.759	0.151	-	-

Table S6. Overall (all stations pooled) decline and recovery trajectories of coral abundance during the study period. Post-hoc tests were used to compare mean abundance values at the end of the study (2014) to initial values (2003). This analysis was not made for coral recruits as no significant temporal variability was detected.

Coral stages/taxa		Decline rate (2003 to 2010)	Recovery rate (2010 to 2014)	2003 vs. 2014 (p-value)
Adults	*Pocillopora*	95%	109%	0.999
	Porites	82%	50%	0.145
	Acropora	99%	14%	0.098
	Montipora	92%	70%	0.985
	All 19 genera	85%	57%	0.932
Juveniles	*Pocillopora*	47%	371%	< 0.001
	Porites	28%	108%	0.226
	Acropora	68%	3%	0.099
	Montipora	10%	100%	0.809
	All 19 genera	53%	173%	< 0.001

Table S7. List of the coral genera recorded within transects established for percent cover estimates (% cover), and within belt-transects for juvenile and adult abundance surveys.

Genera	% cover	Juveniles	Adults
Acanthastrea	+		+
Acropora	+	+	+
Astreopora	+	+	+
Cyphastrea	+	+	+
Favia	+	+	+
Fungia	+	+	+
Gardineroseris	+	+	+
Herpolitha	+	+	+
Leptastrea	+	+	+
Leptoseris	+	+	+
Lopophyllia	+	+	+
Montastrea	+	+	+
Montipora	+	+	+
Pachyseris		+	+
Pavona	+	+	+
Pocillopora	+	+	+
Porites	+	+	+
Psammocora	+	+	+
Sandalolitha	+	+	+
